# A Novel Brazilian Isolate Expands the Genomic and Phenotypic Characterization of *Chlorovirus americanus*

**DOI:** 10.3390/v18101119

**Published:** 2026-10-09

**Authors:** Isadora L. J. Canto, Clécio A. C. Filho, Lethícia R. Henriques, João Victor R. P. Carvalho, Maurício T. Lima, Felipe C. M. Iani, Amanda Haisi, João P. Araújo Júnior, Rodrigo A. L. Rodrigues

**Affiliations:** 1 Departamento de Microbiologia, Universidade Federal de Minas Gerais, Belo Horizonte 31270-901, MG, Brazil; isadoralcanto@gmail.com (I.L.J.C.); clecioalonso@gmail.com (C.A.C.F.); henriques.lethicia@gmail.com (L.R.H.); jvrodrigues934@gmail.com (J.V.R.P.C.); 2 Departamento de Ciências Agrárias, Universidade Federal de São João del-Rei, Sete Lagoas 95701-970, MG, Brazil; 3 Serviço de Virologia e Riquetsioses, Fundação Ezequiel Dias, Belo Horizonte 30510-010, MG, Brazil; maurili15@hotmail.com (M.T.L.); felipe.iani@funed.mg.gov.br (F.C.M.I.); 4 Departamento de Ciências Químicas e Biológicas, Instituto de Biociências, Universidade Estadual Paulista Júlio de Mesquita Filho, Botucatu 18618-970, SP, Brazil; amanda.haisi@unesp.br (A.H.); joao.pessoa@unesp.br (J.P.A.J.)

**Keywords:** Phycodnavirus, *Chlorovirus americanus*, biodiversity, aquatic viruses

## Abstract

A novel chlorovirus was isolated from a freshwater sample collected from a Brazilian urban lake. Members of the family *Phycodnaviridae*, the chloroviruses have large linear dsDNA genomes (285–410 kbp) that encode hundreds of proteins and tRNAs. Despite their ecological and evolutionary importance, chloroviruses remain poorly studied, especially in South America. This study aimed to expand the understanding of this viral group by characterizing a new chlorovirus found in Brazil within a species-level framework. Following inoculation of algal cultures with environmental samples, plaque formation was observed 72 h after incubation on a *Chlorella variabilis* lawn. The new isolate was named BR-MG-N02. The virus remained stable across a temperature range of −30 °C to 37 °C, yet was highly sensitive to UV exposure. This virus has a capsid of approximately 160 nm and establishes a large viral factory in the host cytoplasm. Whole-genome hybrid sequencing revealed a 344,634 bp genome, containing 11 tRNAs and 389 protein-coding sequences. Phylogeny and comparative genomic analyses revealed that BR-MG-N02 is a new member of the species *Chlorovirus americanus*. We show that viruses belonging to this species harbor an extensive DNA methylation machinery, accounting for ~5% of their gene content and suggesting a potential for DNA methylation. Using representatives isolates, we present evidence of intraspecific differences in biological traits of these chloroviruses, as for burst size and plaque morphology. Altogether, our results expand current knowledge about the viral diversity in Brazil and provide valuable insights into the biology of giant algal viruses.

## 1. Introduction

Aquatic ecosystems are rich in biodiversity, particularly in the diversity and abundance of viruses, with estimates indicating more than 10^31^ viral particles around the planet [1]. Among the most abundant groups in these environments are the so-called giant viruses, especially those associated with protists [2]. The term giant virus was first proposed in the late 1990s to describe a virus that infects Chlorella-like algae, known as Paramecium bursaria Chlorella virus 1 (PBCV-1) [3]. This virus has a complex pseudoicosahedral capsid approximately 190 nm in diameter, composed of multiple proteins and an internal lipid membrane that encloses a linear double-stranded DNA (dsDNA) genome [4]. Until the early 21st century, it was considered the virus with the largest genome and the greatest number of genes known in the virosphere, with 331 kbp and over 400 genes, earning it the designation of a giant virus. This concept was significantly expanded after the discovery of mimiviruses and other giant amoeba-infecting viruses, such as pandoraviruses, and is now commonly used to refer to members of the phylum *Nucleocytoviricota* [5,6].

The discovery and subsequent extensive studies of PBCV-1 led to the establishment of the family Phycodnaviridae, which includes several giant viruses that infect algae. The most studied group within this family is the genus *Chlorovirus* [7]. Chloroviruses infect algae in the family Chlorellaceae, which establish symbiotic relationships with protists (e.g., protozoa and heliozoans) and metazoans (e.g., cnidarians), resulting in a complex network of ecological interactions in freshwater environments [8,9,10]. These viruses replicate their genomes in the host cell nucleus and form large viral factories in the cytoplasm, where new particles are assembled and later released through cell lysis [11]. The genomes of currently known chloroviruses range from 285 to 410 kbp, encoding hundreds of proteins and tRNAs, which form an islet in the central region of the genome [12,13]. Phylogenomic analyses have identified three groups of chloroviruses, which we refer to as *Alphachlorovirus*, *Betachlorovirus*, and *Gammachlorovirus* [12,14]. This classification aligns with the traditional grouping of chloroviruses based on their host specificity: *Chlorella variabilis* NC64 and *C. variabilis* Syngen 2-3 (*Alphachlorovirus*); *Micractinium conductrix* Pbi (*Betachlorovirus*); and *Chlorella heliozoae* SAG3.83 (*Gammachlorovirus*) [14,15].

Chloroviruses have been isolated from various locations around the world, particularly in the USA and Europe, but little is known about their presence and diversity in the Global South. Genomic analyses of 36 isolates from different chlorovirus groups revealed an open pan-genome, suggesting there is still much to uncover regarding the genetic and biological diversity of these viruses [16]. Brazil contains approximately 12% of the planet’s surface freshwater, yet studies on giant algal viruses in the country remain scarce. So far, only one chlorovirus was isolated and characterized in the country, named Chlorovirus BR-MG-N01 [17]. In this study, we explored Brazilian aquatic environments in search of chloroviruses, aiming to expand our understanding of their biology and genomics in the Southern Hemisphere and to gain deeper insights into the diversity and evolution of these ecologically important viruses.

## 2. Materials and Methods

### 2.1. Host and Virus Acquisition

For the viral prospecting study, we used microalgae from the species *Chlorella variabilis* NC64A, *Chlorella variabilis* Syngen 2-3, *Chlorella heliozoae* SAG3.83, and *Micractinium conductrix* Pbi. *Chlorella* species were cultured in modified Bold’s Basal Medium (MBBM) supplemented with tetracycline at 10 µg/mL, while *Micractinium conductrix* was maintained in FES medium. Subcultures were performed weekly at a 1:10 ratio. Cultures were incubated at 25 °C under continuous light in temperature-controlled incubators. The chloroviruses isolates AR158 and NY2A were used as viral controls in the experiments. All algal strains and chloroviruses AR158 and NY2A were kindly provided by Professor James Van Etten from the Nebraska Center for Virology, University of Nebraska–Lincoln, USA.

### 2.2. Virus Isolation

Environmental water samples were collected from various regions of Brazil, primarily in the state of Minas Gerais, by collaborators from the Virus Laboratory at the Institute of Biological Sciences, Federal University of Minas Gerais, Brazil. All samples were kindly provided for this project and stored at 4 °C for later use. Samples were filtered through 0.45 µm nitrocellulose membrane filters, and 1 mL of each was used to infect cultures of the selected microalgae, all in the exponential growth phase at a concentration of 1 × 10^8^ cells/mL. To confirm the presence of viral infection, the cytophatic effect in alga cultures were observed and then plaque assays were performed on Petri dishes using the double-layer method. Each plate received a bottom layer of 15 mL of medium supplemented with 1.5% agar, followed by a top layer containing 6 mL of medium with 0.75% agar, 1 mL of the sample, and 1 mL of host cells at a concentration of 1 × 10^9^ cells/mL. Plates were incubated for up to 14 days, monitoring for the appearance of lysis plaques.

From a positive sample, a single lysis plaque was collected and transferred to a microtube containing 1 mL of PBS. The tube was vortexed and centrifuged, and 100 µL of the supernatant was used to inoculate 5 mL of *Chlorella variabilis* NC64A culture in the exponential growth phase. The infection was incubated on a shaker at 150 rpm for 7 days. After incubation, the culture was centrifuged, and 100 µL of the supernatant was serially diluted and titrated using the double-layer method in 6-well plates. Once the viral titer was determined, a low multiplicity of infection (MOI 0.01) was carried out in 400 mL of *Chlorella variabilis* NC64A (1 × 10^8^ cells/mL), maintained under constant light and agitation at 25 °C for 7 days. The resulting lysate was centrifuged at 4000× *g* for 10 min, and the supernatant was filtered through a 0.22 µm membrane filter and ultracentrifuged at 24,000× *g* for 60 min at 4 °C. The viral pellet was resuspended in 5 mL of Tris-HCl buffer (pH 7.2) and subsequently purified by centrifugation in a 10–40% sucrose gradient at 36,000× *g* for 20 min. The purified viral band was collected, and the viral titer was determined using the double-layer assay in 6-well plates.

### 2.3. Virus Stability Assays

The isolated virus was subjected to tests to assess its stability under different temperatures and after exposure to ultraviolet (UV) radiation. For the temperature assays, 100 µL aliquots of the purified virus were placed in microtubes and exposed to various temperatures for 10 min. The −80 °C and −30 °C treatments were conducted using ultralow freezers; samples at 4 °C were stored in a refrigerator, while those at 37 °C, 56 °C, and 100 °C were incubated in a thermoblock. For UV radiation assays, 6-well plates containing 100 µL of purified virus per well were exposed to a UV-C light source, 15 cm distant, inside a biological safety cabinet. Viral samples were collected at different time points (1, 2, 3, 4, 5, and 10 min). In both assays, the treated viral samples were serially diluted and titrated using the double-layer plaque assay in 6-well plates. All experiments were done in biological duplicates.

### 2.4. Viral Replication and Host Range Assays

To assess the growth profile of the new viral isolate, compared to the viruses of the same species (AR158 and NY2A), infections at an MOI of 0.01 were performed using 3.6 × 10^8^ *Chlorella variabilis* NC64A cells. Infections were maintained on a shaker at 150 rpm for 24 h. After incubation, cultures were centrifuged at 4000× *g* for 10 min, and the supernatant was collected, serially diluted, and titrated using the double-layer plaque assay in 6-well plates. We also evaluated host range of the isolated virus. For this, 10 µL of purified virus was added to Falcon tubes containing 5 mL of microalgal cultures from the species *Chlorella heliozoae*, *Micractinium conductrix*, and *Chlorella vulgaris*. Tubes were maintained under agitation for 7 days, and the cultures were monitored daily for potential cytopathic effects.

### 2.5. Transmission Electron Microscopy Analysis

The purified viral isolate was analyzed at the UFMG Microscopy Center for transmission electron microscopy (TEM) to assess the morphology and replication cycle. For negative staining, 100 µL of the viral suspension was treated with uranyl acetate and placed onto copper grids for observation. Additionally, embedded samples were prepared from an infection at MOI 1 in 5 mL of *Chlorella variabilis* NC64A culture (total of 5 × 10^8^ cells). The infection was maintained on a shaker at 150 rpm for 8 h. After this period, the culture was centrifuged at 4000× *g* for 10 min, and the pellet was resuspended in 1.5 mL of Karnovsky solution (2% paraformaldehyde and 2.5% glutaraldehyde), sonicated briefly, and kept under agitation at 150 rpm for 4 h. The material was then washed three times with 0.1 M phosphate buffer (pH 7.3) and sent to the Microscopy Center, where it was post-fixed with osmium tetroxide, embedded in resin, sectioned, and contrasted. Both preparations were visualized using a Tecnai G2-12-Spirit BioTwin FEI 120 kV transmission electron microscope (Thermo Fisher, Waltham, MA, USA). Images were processed and analyzed using the ImageJ software v1.54g.

### 2.6. Genome Sequencing, Assembly, and Annotation

The purified virus was sequenced using the Illumina MiSeq i100 with a paired-end 2 × 300 bp library, prepared with the Illumina DNA Prep kit (Illumina Inc., San Diego, CA, USA). Raw reads were analyzed using FastQC for quality assessment and trimmed to remove low-quality bases with Trimmomatic [18]. The genome was also sequenced using MinION technology. For that, viral DNA was sheared to a target fragment size of ~6 kbp using a Covaris g-TUBE (Covaris, Woburn, MA, USA). Samples were centrifuged twice, first with the screw-cap up, then inverted with the screw-cap down, at 11,000 RPM for 30 s, following the manufacturer’s protocol. Approximately 1 µg of sheared DNA was used as input for library preparation, performed using the Ligation Sequencing Kit (SQK-LSK109; Oxford Nanopore Technologies, Oxford, UK) in combination with the Native Barcoding Expansion 1-96 kit (Oxford Nanopore Technologies), following the manufacturer’s protocol. A total of 30 ng of library was loaded onto an R9.4.1 flow cell (FLO-MIN106; Oxford Nanopore Technologies) and sequenced on a MinION device (Oxford Nanopore Technologies) for 18 h. Basecalling and demultiplexing were performed using Guppy v6.0.1.

Hybrid assembly was performed using the SPAdes program (version 3.15.5) [19]. Open reading frames (ORFs) were predicted using Prodigal (version 2.6.3) [20], and tRNAs were identified using the default parameters of ARAGORN [21]. ORFs were annotated using BLASTp (NCBI nr database accessed at 18 August 2026), HHpred (accessed at 18 August 2026, available at https://toolkit.tuebingen.mpg.de/tools/hhpred/), and InterProScan (accessed at 18 August 2026, available at https://www.ebi.ac.uk/interpro/search/), following the strategy described elsewhere [12]. Functional categorization of the predicted proteins was performed based on Orthologous Groups of Nucleo-Cytoplasmic Viruses [22].

### 2.7. Genomic and Phylogenetic Analysis

The genome was visualized in circular format using Proksee [23]. Average Nucleotide Identity (ANI) analysis was performed with FastANI [24], using the complete genomes of eight chlorovirus isolates. Synteny analysis was conducted using DiGAlign [25]. Clusters of orthologous groups of genes (COGs) were constructed with OrthoFinder using default parameters [26]. Methyltransferases COGs were constructed using ProteinOrtho6 with the following parameters: e = 1 × 10^−5^; cov = 50; identity = 50 [27]. Pan-genome evolution was assessed based on the stepwise addition of isolates, and the COG-sharing pattern was visualized using Gephi 0.10 [28]. Pan-genome and methyltransferase-related networks were generated using ForceAtlas, a force-directed layout algorithm implemented in Gephi, and were minimally edited manually to improve the visualization of singleton nodes.

For phylogenetic reconstruction, amino acid sequences of the chlorovirus DNA polymerase B family genes were aligned using MUSCLE 5.3 with default parameters [29] and subsequently trimmed with TrimAl 1.5.1 to remove misaligned regions [30]. The phylogenetic tree was constructed using IQ-TREE2 2.4.0 with the maximum likelihood method and 1000 bootstrap replicates [31], employing the best-fit evolutionary model selected by ModelFinder 2.4.0 [32]. The resulting tree was visualized using iTOL v6 [33].

## 3. Results

### 3.1. A New Chlorella Virus Found in the Waters of Minas Gerais, Brazil

**Figure 1:**
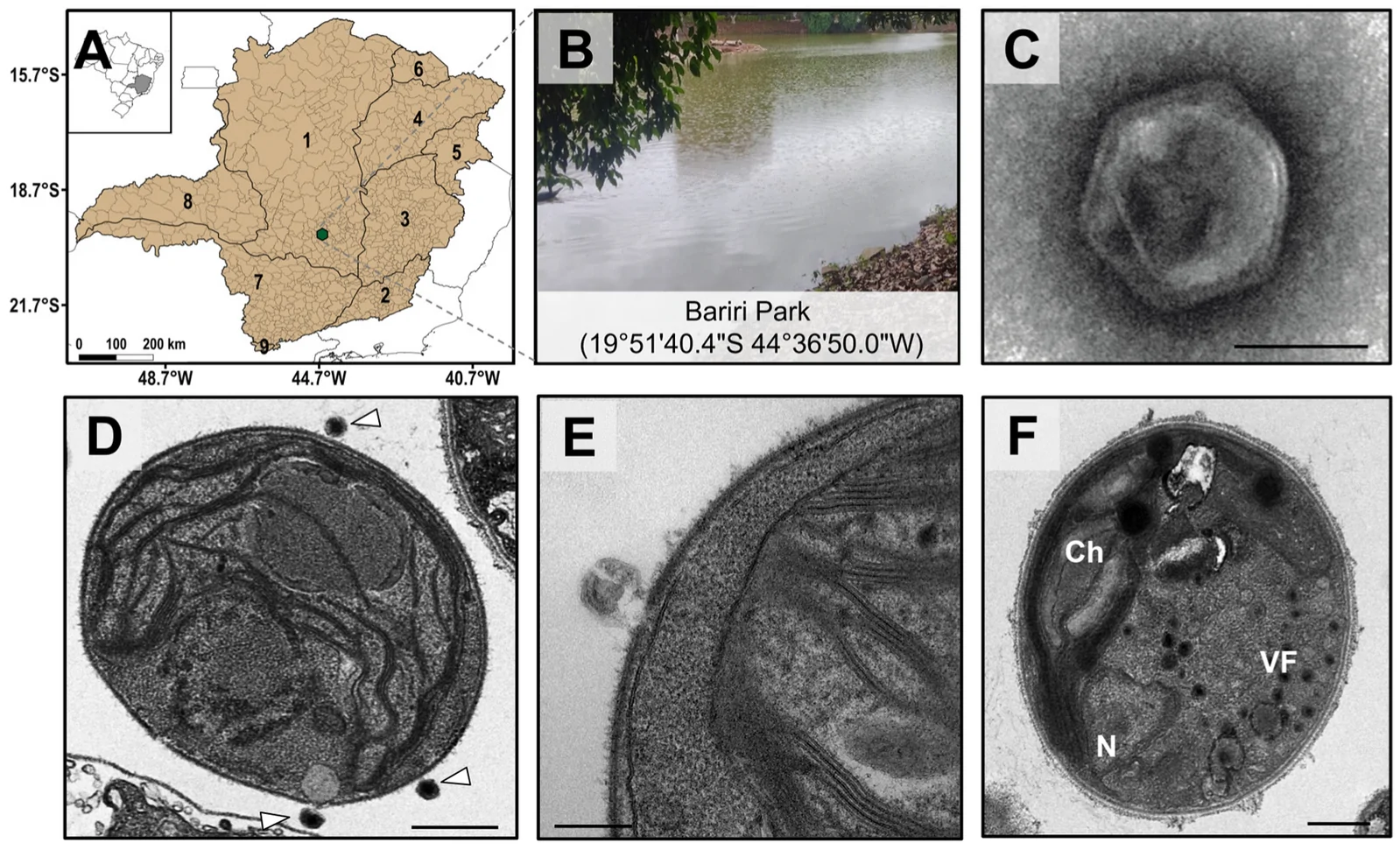
Isolation and morphological characterization of Chlorella virus BR-MG-N02. (**A**) Isolation site of a new chlorovirus. The map was created with QGIS 4.2 software and is divided based on the main river basins present in the state, named [1] São Francisco River; [2] Pardo River; [3] Jequitinhonha River; [4] Mucuri River; [5] Doce River; [6] Paraíba do Sul River; [7] Paranaíba River; [8] Grande River; and [9] Piracicaba River. The area highlighted in green indicates the Bariri Municipal Park, located within the São Francisco River basin, where the BR-MG-N02 was isolated. (**B**) Representative picture of the lake from where the virus was isolated, including geographic coordinates. (**C**) Negative staining transmission electron microscopy (TEM) of chlorella virus BR-MG-N02, revealing an icosahedral particle with inner membrane. (**D**) Viral particles attached to the host cell wall (white arrows). (**E**) Cell wall degradation upon viral attachment. (**F**) A large viral factory is established in the cell cytoplasm, where the assembly of new viral particles occurs. N: Nucleus, Ch: Chloroplast, VF: Viral factory. Scale bars: (**C**) 100 nm; (**D**,**F**) 500 nm; (**E**) 200 nm.

Water samples collected in September 2024 were filtered and inoculated into microalgae cultures for up to 14 days to observe cytopathic effects. In a sample collected from a lake in Bariri Municipal Park, in the city of Pará de Minas, Minas Gerais state (Figure 1A,B), we observed the presence of lysis plaques in *C. variabilis* NC64A after 72 h of infection, indicating viral activity. The lysis plaque was collected and inoculated into a new algal culture for viral propagation and subsequent rounds of plaque purification. We named the new isolate BR-MG-N02. Once purified, a fresh algal culture was infected, and complete cell lysis and total culture clearance were observed within 7 days post-infection, a similar effect to that of chlorovirus BR-MG-N01 [17]. The BR-MG-N02 isolate forms lysis plaques of approximately 2 mm in diameter after 7 days of infection, which is considered medium-sized when compared to other known chloroviruses [34]. The new isolate did not cause cytopathic effects in any other algal species tested, suggesting a narrow host range. Transmission electron microscopy revealed an icosahedral particle approximately 160 nm in diameter, with an internal layer surrounding the genome, consistent with the inner membrane of chloroviruses (Figure 1C). The new isolate demonstrated good thermal stability between −30 °C and 37 °C, maintaining titers above 10^9^ PFU/mL. However, the titer dropped by 2 logs when stored at −80 °C and by approximately 5 logs after exposure to 56 °C for 10 min (Figure S1A). The virus was completely inactivated at 100 °C. When exposed to UV radiation, we observed a gradual decrease in viral titer between 0 and 5 min of exposure, dropping from 3.4 × 10^9^ PFU/mL to 1 × 10^3^ PFU/mL, a 99.9999% reduction in infectious particles (Figure S1B). Complete inactivation occurred after 10 min of UV exposure. Viral particles attach to the cell wall, which is degraded establishing a portal through where the virion content is released (Figure 1D,E). Upon entering the host cell, BR-MG-N02 establishes a large viral factory in the cytoplasm, where new viral particles are assembled (Figure 1F). The host nucleus and chloroplast remain unaltered. During the later moments of the infection, the cell wall is damaged and viral particles are released through cell lysis.

### 3.2. The Brazilian Isolate Is a New Member of the Species Chlorovirus americanus

**Figure 2:**
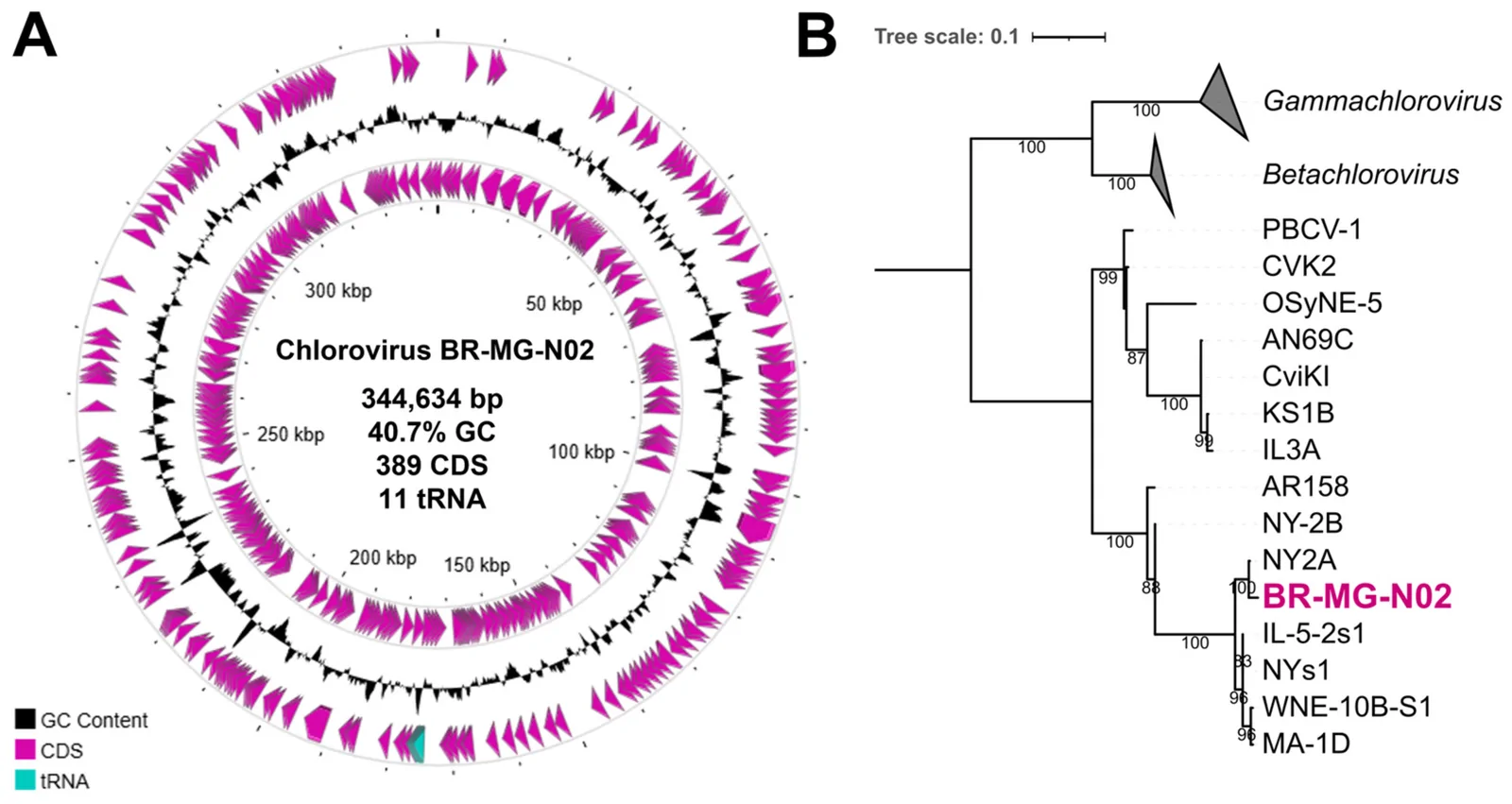
Chlorovirus BR-MG-N02 genome and phylogeny. (**A**) Genomic map of the isolate BR-MG-N02 showing the CDSs in pink, tRNA in cyan, and GC content distribution in black ring. General genomic features are included in the center of the image. (**B**) Phylogenetic tree based on DNA polymerase B family gene, including all three groups of chloroviruses, evidencing the position of isolate BR-MG-N02. The tree was rooted using betachloroviruses and gammachloroviruses as outgroups. Scale bar represents rate of amino acid substitution.

The genome of the new isolate was sequenced using the Illumina paired-end and long-read MinION methods. *De novo* hybrid assembly resulted in a single scaffold of 344,643 bp with a GC content of 40.7% (Figure 2A; Table S1). BLASTn (NCBI nr database accessed at 12 July 2025) analysis identified *Chlorovirus americanus* isolates (e.g., AR158 and NY2A) as the best hits. The genome of the BR-MG-N02 isolate has a coding density of 90.8%, with 389 coding sequences (CDSs) homogeneously distributed between the forward and reverse strands. Genes have an average GC content of 40.8%, ranging from 24.8% to 58.4%, and we found 11 tRNAs, all located on the forward strand (Figure 2A). In general, chloroviruses exhibit a tRNA islet positioned near the center of the genome [13]. In all *Chlorovirus americanus* isolates, the central genome positioning of the tRNA islet is maintained (Table S2). Among the 11 tRNAs encoded by BR-MG-N02, eight amino acids are represented, including one duplication each for asparagine, leucine, and lysine, the latter two featuring distinct anticodons (Table S2). The tRNA repertoire of BR-MG-N02 is similar to that of other members of the species, with no unique tRNA identified. Based on the DNA polymerase B family gene, a well-established phylogenetic marker for viruses in the phylum *Nucleocytoviricota* [6], we confirmed that BR-MG-N02 clusters with other isolates of the species *Chlorovirus americanus* (subgenus *Alphachlorovirus*) with strong statistical support (bootstrap = 100) (Figure 2B).

**Figure 3:**
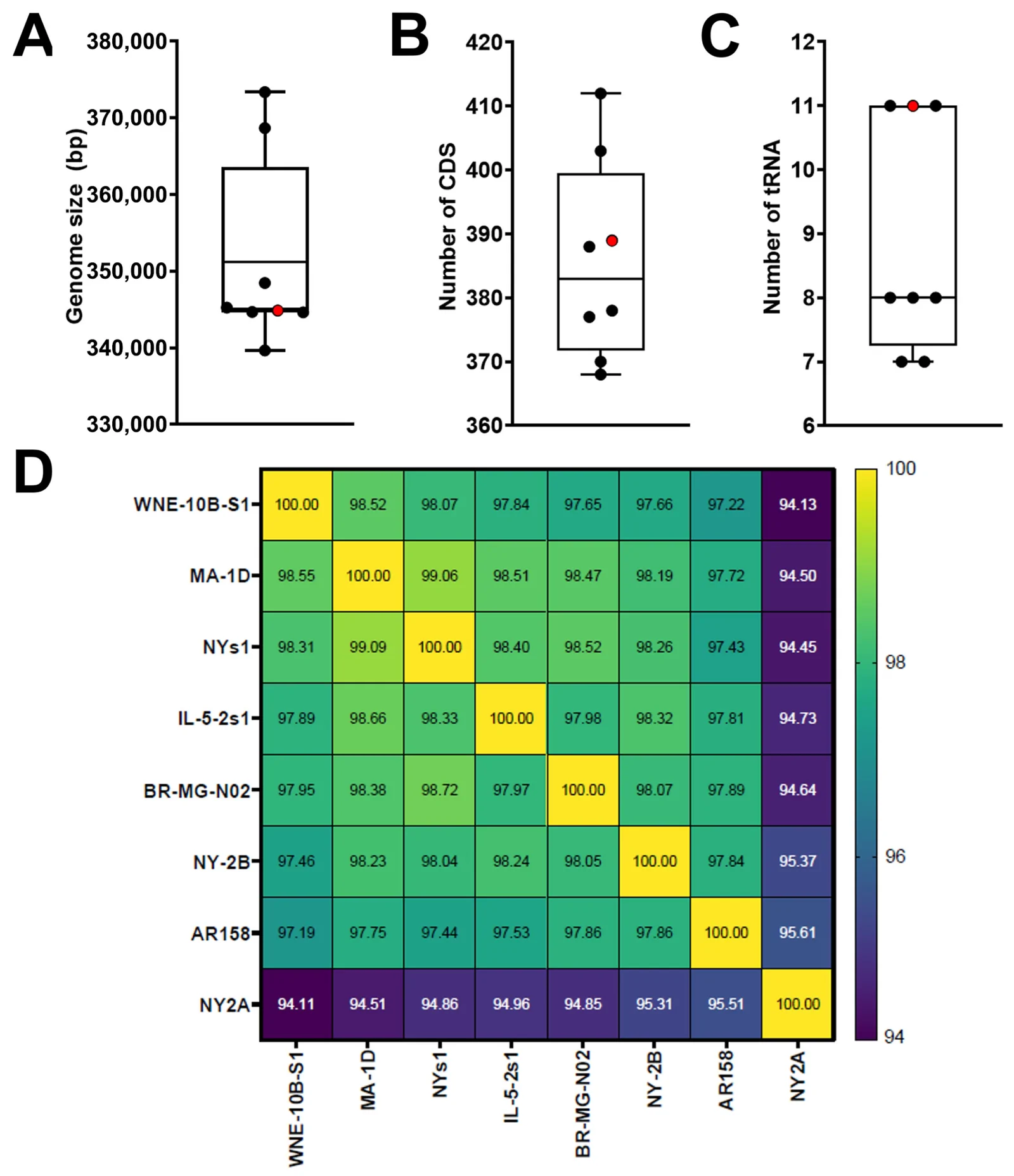
Comparative genomic features of isolates of *Chlorovirus americanus.* Boxplots of general genomic features of Chlorovirus americanus isolates, named (**A**) genome size; (**B**) CDS count; (**C**) tRNA count. The chlorovirus BR-MG-N02 is highlighted as red dots; (**D**) Heatmap showing average nucleotide identity values including all eight *Chlorovirus americanus* isolates. Scale bar indicates ANI values ranging from 94 to 100%.

BR-MG-N02 has the second smallest genome reported within its species, whose genome sizes average approximately 351 kbp, ranging from 338 kbp to 373 kbp (e.g., WNE-277 10B-S1) (Figure 3A; Table S1). A total of 389 coding sequences (CDSs) longer than 40 amino acids were predicted, a gene amount slightly above the species average (385 CDSs) (Figure 3B; Table S1). Interestingly, isolate NY2A has the highest number of predicted genes (*n* = 412), despite having a genome smaller than the species average (368 kbp). Although BR-MG-N02 has one of the smallest genomes among known *C. americanus* isolates, it encodes more genes than half of them (Table S1). Additionally, 11 tRNAs were predicted for BR-MG-N02, above the species average of 8 tRNAs and equal to WNE-10B-S1 and MA-1D (Figure 3C; Table S1). Average Nucleotide Identity (ANI) analysis revealed values above 94% shared with other *C. americanus* isolates, corroborating the phylogenetic placement of BR-MG-N02 as a new member of the species (Figure 3D). Among the isolates, NY2A was the most divergent, sharing an average of 95% identity with the others, whereas BR-MG-N02 showed approximately 98% identity with its conspecifics. When compared to PBCV-1 (*Chlorovirus vanettense*), BR-MG-N02 shared an average genomic identity of 85.8%.

### 3.3. Genomic Analyses of BR-MG-N02 Isolate Reveal an Extensive DNA Methylation Machinery in Chlorovirus americanus

**Figure 4:**
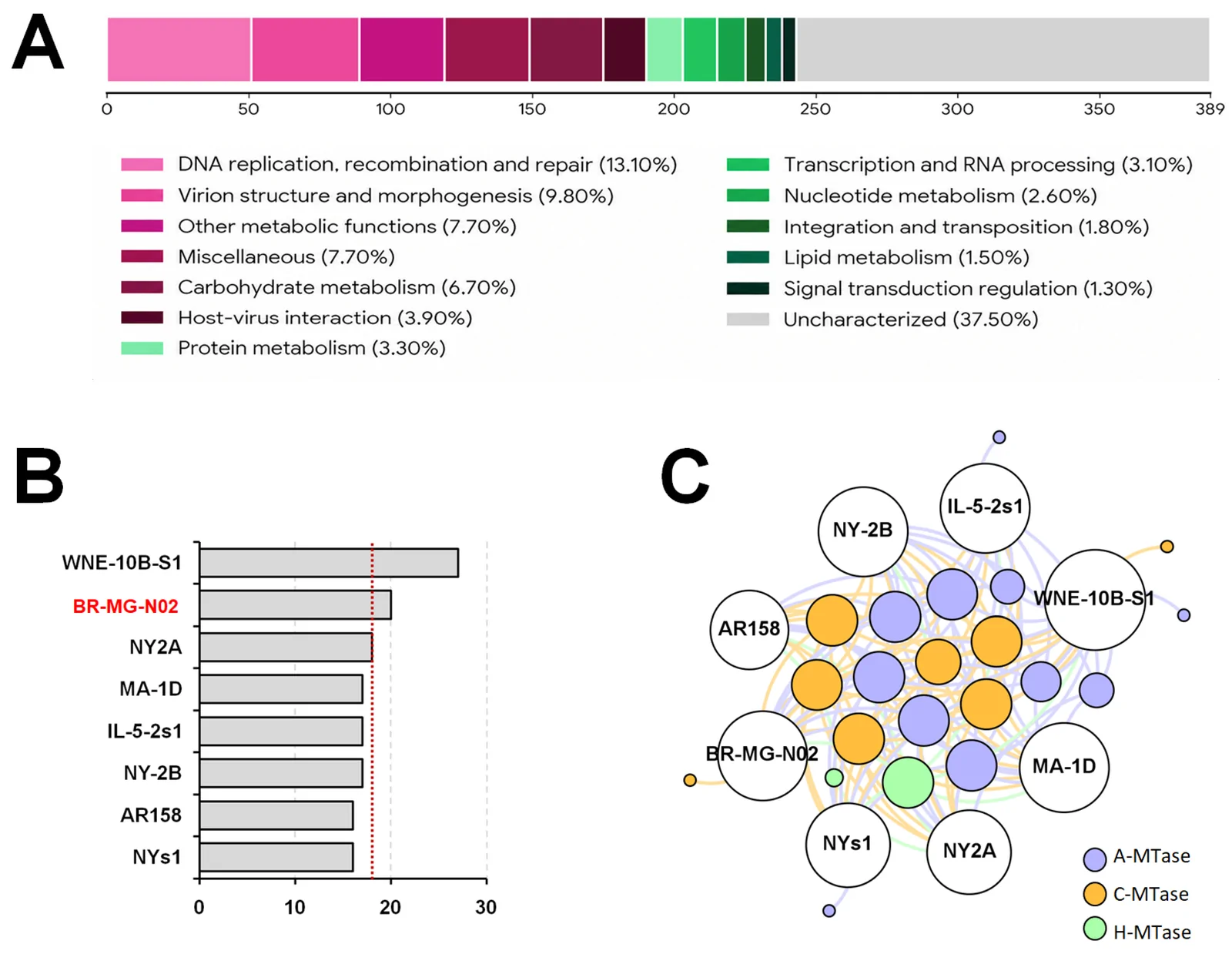
Genome annotation of Chlorovirus BR-MG-N02. (**A**) Functional categorization of genes predicted in BR-MG-N02 genome. (**B**) Abundance of methyltransferases (MTases) in isolates of *Chlorovirus americanus*. Red line in the column chart represents the mean value of genes found in the viral genomes. (**C**) MTases gene-sharing within isolates of *Chlorovirus americanus*. In the network, the node sizes are proportional to the degree of connection. White nodes represent the viruses and are labeled with the isolates’ names, while colored nodes represent different MTases, named adenine-specific (A-MTase), cytosine-specific (C-MTase), and histone-specific (H-MTase).

Of the 389 predicted CDSs, over one-third (*n* = 137; 35.3%) have no known function (Figure 4A). Three of these are ORFans, with no detectable similarity to any known protein sequences in public databases. The identification of such novel genes, albeit limited in number, suggests the existence of unexplored genetic diversity among chloroviruses. Among the annotated genes, we identified a wide array of enzymes involved in carbohydrate metabolism, including glycosyltransferases, chitinases, chitosanases, and alginate lyases, which have also been reported in other chloroviruses. Additionally, genes related to lipid and protein metabolism were found, including lipid hydrolases and E3 ubiquitin ligases, enzymes responsible for the degradation of lipids and proteins, respectively. A total of 11 genes involved in transcriptional regulation or RNA modification were identified, including transcription factors (e.g., TFIIB, TFIID, and TFIIS), RNase III, and an mRNA capping enzyme. No RNA polymerase subunit was detected, consistent with observations in other chloroviruses [12,15,35]. Numerous genes involved in virion structure and morphogenesis were also identified (Figure 4A), including the major capsid protein (Vp54), several minor capsid proteins homologous to those found in PBCV-1 [4], and the DNA packaging ATPase, a gene conserved across the phylum Nucleocytoviricota and implicated in genome packaging [36,37].

Among the functional categories, the most abundant was DNA replication, recombination, and repair, with 51 genes (13.11%) involved in these processes (Figure 4A). It includes genes commonly found in giant viruses, such as DNA polymerase, DNA topoisomerase, DNA primase, and helicases, as well as various endonucleases, including those with GIY-YIG catalytic domains, enzymes previously described in other chloroviruses. Notably, we observed a large number of methyltransferases (MTases) dispersed throughout the BR-MG-N02 genome. A total of 20 enzymes involved in methylation of adenine, cytosine, and histones were identified, accounting for approximately 5% of the isolate’s gene content. MTases were also found in other isolates of *C. americanus*, with an average of 18 MTase genes per genome (Figure 4B). Only isolate WNE-10B-S1 harbors more MTases than BR-MG-N02, with 27 genes distributed across its genome. Proportionally, we did not observe major differences among the isolates, with MTase-related genes comprising between 4.12% and 6.70% of their total gene content. Most of the MTases are orthologous, forming conserved gene clusters among the isolates. However, we identified unique MTases in four isolates, including a cytosine-specific DNA methyltransferase in BR-MG-N02 (Figure 4C).

### 3.4. Chlorovirus americanus Has Conserved Genomic Features, but Differences in Biological Traits

Synteny analysis revealed a high conservation of gene organization among *Chlorovirus americanus* isolates, with a strong degree of gene identity, reinforcing the results observed in the ANI analysis (Figure S2). Minor variations are present in the genomes of isolates IL-5-2s1 and MA-1D, both maintaining a high degree of identity (>95%). Although isolate NY2A shows a lower overall similarity to the other isolates, its genomic organization remains well conserved (Figure S2). The terminal regions of the genomes are less conserved relative to the rest of the genome, exhibiting approximately 85% identity in some isolates (e.g., BR-MG-N02 and NY-2B).

**Figure 5:**
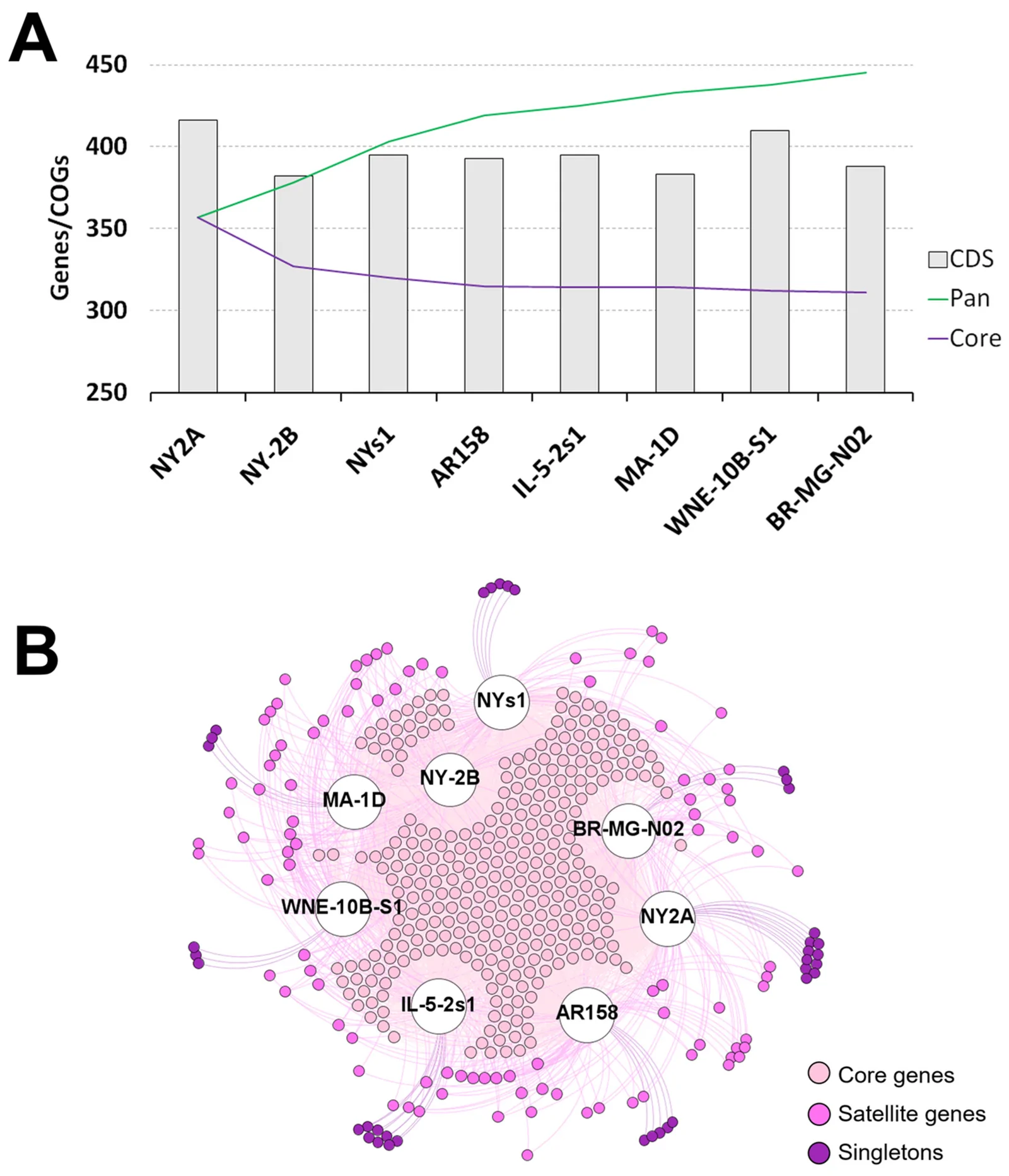
Pan-genome evolution and COG-sharing pattern of *Chlorovirus americanus*. (**A**) Pangenome evolution including all eight isolates that belong to the species *Chlorovirus americanus*. Columns indicate the amount of CDS predicted in each genome. Green curve indicates the increase in the pan-genome, and purple curve refers to the decrease in the core genome with the addition of new isolates to the species. (**B**) Bipartite network including viruses (white nodes) and the shared COGs (colored nodes). The node sizes are proportional to the degree of connection.

After defining the species to which the Brazilian isolate belongs, we evaluated its impact on the evolution of the *Chlorovirus americanus* pangenome and the pattern of clusters of orthologous genes (COGs) shared among viral isolates (Figure 5). A total of 3163 predicted genes from the eight isolates were grouped into 445 COGs, constituting the species’ pangenome. As we added isolates, we observed a marginal increase in COGs, ranging from 1.15% to 6.61% depending on the isolate, reaching a cumulative increase of 24.64% (Figure 5A). Conversely, we observed a slightly more pronounced reduction in shared COGs among the isolates (core genome), followed by stabilization of the curve (Figure 5A). Comparing the first two isolates analyzed, NY2A and NY-2B, there was an 8.4% reduction in the core genome. The addition of the remaining isolates resulted in a mild decrease, between 2.1% and 0%, indicating stabilization. Overall, we calculated a 12.9% reduction in the core genome of *Chlorovirus americanus* considering all isolates of the species. The inclusion of BR-MG-N02 contributed to a 1.59% increase in the number of COGs and only a 0.32% decrease in the core genome. Of the 445 COGs identified, 311 (69.9%) are shared by all isolates, constituting the core genome; 97 (21.8%) are shared by two or more isolates, constituting the accessory genome; and 37 (8.3%) are singletons (Figure 5B). Our analysis identified three singletons unique to the new Brazilian isolate (the three predicted ORFans), a number comparable to other isolates, except for NY-2B, which had no unique COGs, and NY2A, which contained 10 unique COGs (Figure 5B).

**Figure 6:**
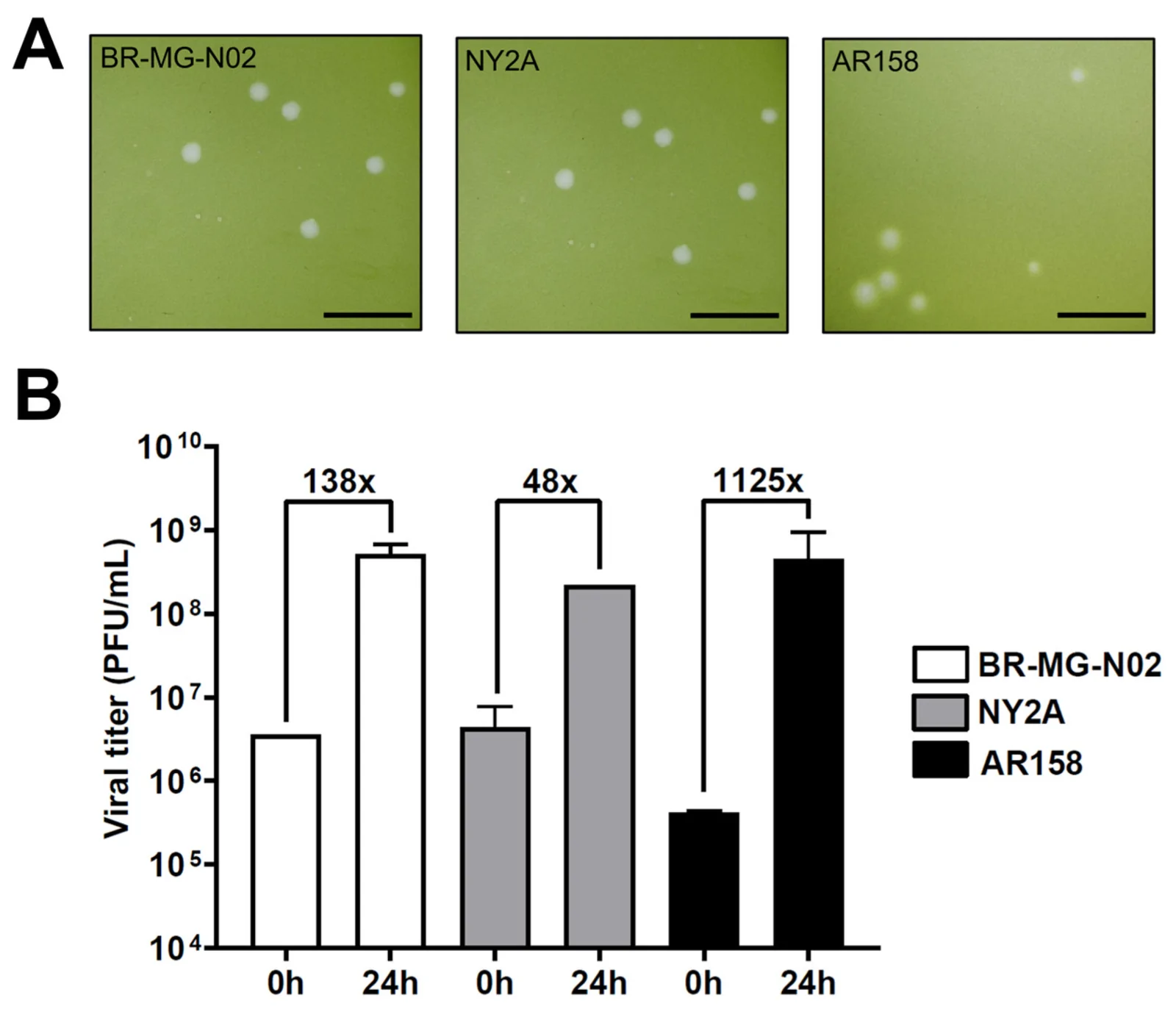
Biological features of *Chlorovirus americanus* isolates. (**A**) Plaque morphology of isolates BR-MG-N02, NY2A, and AR158 in Chlorella variabilis NC64A lawns. Scale bars: 1 cm. (**B**) Viral yield within 24 h of infection of three isolates of *Chlorovirus americanus*. Titers are expressed as plaque-forming unit (PFU) per mL.

Curiously, despite the conservative genomic features, we observed differences in biological traits of different chloroviruses isolates, including plaque morphology and burst size (Figure 6). Chloroviruses BR-MG-N02, NY2A, and AR158 form lysis plaques of ~2 mm in diameter on average in *C. variabilis* NC64A lawns (Figure 6A), but small variations can be observed. Different plaque morphology, especially considering the plaque size, can occur for a chlorovirus even after many rounds of plaque purification. Other isolates of the species exhibit regular plaques of ~1 mm in diameter (James Van Etten, personal communication). Although the plaque morphology seems similar among different isolates, the burst size exhibit considerable differences. After 24 h of infection, we observed a 2-log_10_ increase in BR-MG-N02 particles in the supernatant, whereas AR158 exhibited a 3-log_10_ increase (Figure 6B). This increase was higher than that observed for NY-2A (1.5-log_10_), but relatively lower than that observed for AR158 (3-log_10_) (Figure 6B). These results indicate that chloroviruses belonging to the same species may possess conserved genomic characteristics yet exhibit distinct biological traits. Further investigation into the biology of these viruses will be necessary to better understand the extent of virus intraspecific variation.

## 4. Discussion

Chloroviruses were discovered over 40 years ago, and much of our current knowledge is based on studies of PBCV-1, which for years was considered the prototype of the genus *Chlorovirus* [for a detailed review, see Van Etten et al., 2020—[38]]. The isolation of new viruses has enabled progressive advances in understanding the diversity and evolution of this group of aquatic viruses, revealing several species that cluster into three well-defined phylogenetic clades [12,35]. These viruses have been isolated from freshwater samples in various regions worldwide; however, the full extent of chlorovirus genomic and biological diversity remains far from fully characterized [12,15,35,39]. In Brazil, only one isolate has been isolated and characterized to date [17]. Given Brazil’s renowned biodiversity and its possession of over 10% of the planet’s surface freshwater, there is a significant knowledge gap regarding the presence, diversity, and ecology of chloroviruses both in Brazil and globally. In this work, we contribute to filling this gap by isolating and characterizing a new chlorovirus from the waters of Minas Gerais, one of Brazil’s largest states.

The chlorovirus BR-MG-N02 presents a hexagonal particle with a putative inner membrane of approximately 160 nm in diameter, slightly smaller than that described for other chloroviruses [4,40]. This observation is based on negative-staining TEM, and variations may be expected due to the use of chemical reagents that can affect the virion structure. Nevertheless, we expect that its structure is highly similar to that of PBCV-1, as we detected homologs of several structural proteins, including the major capsid protein (MCP Vp54 and its variants MCPv1 and MCPv5), the penton protein (P1) and its variant (P1v1), as well as 19 of 22 minor capsid proteins [4]. The three missing proteins (P20, P21, and P23) may be present but remain undetected due to high divergence beyond our search criteria. Furthermore, an internal lipid membrane has been described for chloroviruses, which is important for the viral life cycle [4,11]. In this regard, it is likely that the inner layer observed in negatively stained TEM images corresponds to the viral membrane. Like other chloroviruses, BR-MG-N02 enters the host cell by inducing a pore in the cell wall, possibly facilitated by alginate lyase and other glycosyl hydrolases present in its particle [41,42]. The virus establishes a large viral factory that occupies nearly the entire cytoplasm, where new particles are assembled and subsequently released through cell lysis [11]. We estimate that each infected cell releases between 100 and 200 particles, a burst size comparable to other chloroviruses [37]. We observed no significant alterations in chloroplasts of infected cells, suggesting that this organelle is not exploited during viral replication. Additional studies should be done to evaluate the impact of cell organelles in chloroviruses replication. Interestingly, BR-MG-N02 is resistant to temperature variations between −30 °C and 37 °C during 10 min exposure, but exhibited reduced infectivity when subjected to −80 °C and 56 °C or higher temperatures. However, prolonged freezing caused a significant decline in viral infectivity, indicating that, like PBCV-1, it cannot tolerate extended exposure to very low temperatures [37]. Additionally, the virus is highly sensitive to UV radiation, being completely inactivated after only 10 min of exposure. Additional studies exposing chlorovirus-infected cells to UV radiation may provide insights into this feature.

BR-MG-N02 is the first Brazilian isolate, and the second one found outside the USA, classified as *Chlorovirus americanus*, a species of the subgenus *Alphachlorovirus*. Both phylogenetic and ANI analyses support this classification, following established strategies used for dozens of other chloroviruses [12,34]. Although BR-MG-N02 has the smallest genome of the species, its structural features do not significantly differ from other known chloroviruses. However, analysis of its genetic content revealed a substantial number of genes encoding methyltransferases, which constitute more than 5% of its gene repertoire, suggesting a highly methylated genome. Methyltransferases have been detected in other chloroviruses, and liquid chromatography analyses have shown wide variation in methylation levels across viral genomes, with the NY2A isolate exhibiting up to 45% methylated cytosines and 37% methylated adenines [43]. We identified 18 MTases in NY2A and 20 in BR-MG-N02, suggesting a comparable methylation level in the Brazilian isolate. The extent of methylation in BR-MG-N02 and other *Chlorovirus americanus* isolates remains to be determined to establish whether this is a species wide or an isolate-specific trait. For PBCV-1 (*Chlorovirus vanettense*), SMRT sequencing data evidenced that viral adenines are stably methylated [44], and earlier studies suggested at least 2% of cytosines and adenines are methylated [45]. Similar methylation levels have also been reported in some betachloroviruses, with 5-methylcytosine (5mC) ranging from 14% to 43% and N6-methyladenine (6mA) up to 18% [46]. Biochemical characterization of some chlorovirus MTases confirmed that these genes are expressed and that the enzymes methylate the viral genome [47,48]. DNA methylation regulates various biological processes, including gene expression and, in viruses, protection against host defenses [49,50]. In chloroviruses, some MTases are accompanied by restriction endonucleases, forming a restriction-modification system [51]. Further study of the MTases found in chlorovirus BR-MG-N02, especially its unique cytosine-specific methyltransferase, may provide valuable insights into virus–host interactions.

The isolation and characterization of new viruses have uncovered unique features of chloroviruses and fostered progress across virology, biochemistry, and viral biotechnology. Notably, several enzymes encoded by chloroviruses have been characterized and developed into molecular biology tools, including endonucleases, methyltransferases and ligases [37,52]. By isolating a new virus from Brazilian waters, we advance knowledge of the presence and diversity of these viruses, particularly in the Southern Hemisphere. The slight expansion of the species’ pangenome suggests ongoing potential for genetic innovation, especially considering the diverse chlorovirus clades [16]. Given Brazil’s continental scale, exploration for new viruses in different regions promises significant advances in the study of giant algal viruses.

## Data Availability

The genome sequence of *Chlorovirus americanus* isolate BR-MG-N02 was deposited in the NCBI GenBank database with accession number PV975788. The datasets generated and/or analyzed during the current study are available in the GenBank repository.

## Supplementary Materials

The following supporting information can be downloaded at: https://www.mdpi.com/article/10.3390/v18101119/s1, Figure S1. Viral resistance to environmental factors. (A) Temperature resistance of BR-MG-N02; (B) UV radiation sensitivity of BR-MG-N02. Biological assays were performed in duplicate. nd: not detected; Figure S2. Genome synteny of *Chlorovirus americanus*. Viral genomes were aligned, and colored lines represent BLASTn identity values. The name of the isolates and their genome sizes are indicated on the left side of the respective genome representation; Table S1: General information about *Chlorovirus americanus* isolates; Table S2: Diversity of tRNA in *Chlorovirus americanus*.

## Abbreviations

The following abbreviations are used in this manuscript:

dsDNA	Double-stranded DNA
bp	Base pairs
Kbp	Kilobase pairs
tRNA	Transfer RNA
UV	Ultraviolet
DNA	Deoxyribonucleic acid
RNA	Ribonucleic acid
PBCV-1	Paramecium bursaria Chlorella virus 1
ICTV	International Committee on Taxonomy of Viruses
MBBM	Modified Bold’s Basal Medium
PBS	Phosphate-buffered saline
MOI	Multiplicity of infection
TEM	Transmission Electron Microscopy
NCBI	National Center for Biotechnology Information
BLASTn	Basic Local Alignment Search Tool (nucleotide)
BLASTp	Basic Local Alignment Search Tool (protein)
ORF	Open Reading Frame
ORFs	Open Reading Frames
ANI	Average Nucleotide Identity
COG	Cluster of Orthologous Groups
COGs	Clusters of Orthologous Groups
GC	Guanine-Cytosine content
CDS	Coding sequence
PFU	Plaque-forming units
Q30	Phred quality score ≥30
MTase	DNA methyltransferase
RNase III	Ribonuclease III
TFIIB	Transcription Factor IIB
TFIID	Transcription Factor IID
ATPase	Adenosine triphosphatase
MCP	Major Capsid Protein
MCPv1	Major Capsid Protein variant 1
MCPv5	Major Capsid Protein variant 5
P1	Penton protein 1
P1v1	Penton protein variant 1
GIY-YIG	GIY-YIG endonuclease
SMRT	Single Molecule Real-Time sequencing
5mC	5-methylcytosine
6mA	N6-methyladenine

## References

[1] Suttle C.A. (2005). Viruses in the sea. Nature.

[2] Schulz F., Abergel C., Woyke T. (2022). Giant virus biology and diversity in the era of genome-resolved metagenomics. Nat. Rev. Microbiol..

[3] Van Etten J.L., Meints R.H. (1999). Giant Viruses Infecting Algae. Annu. Rev. Microbiol..

[4] Shao Q., Agarkova I.V., Noel E.A., Dunigan D.D., Liu Y., Wang A., Guo M., Xie L., Zhao X., Rossmann M.G. (2022). Near-atomic, non-icosahedrally averaged structure of giant virus *Paramecium bursaria* chlorella virus 1. Nat. Commun..

[5] Abergel C., Legendre M., Claverie J.-M. (2015). The rapidly expanding universe of giant viruses: Mimivirus, Pandoravirus, Pithovirus and Mollivirus. FEMS Microbiol. Rev..

[6] Aylward F.O., Moniruzzaman M., Ha A.D., Koonin E.V. (2021). A phylogenomic framework for charting the diversity and evolution of giant viruses. PLoS Biol..

[7] Van Etten J.L., Duningan D.D., Nagasaki K., Schroeder D.C., Grimsley N., Brussaard C.P.D., Nissimov J.I., Bamford D.H., Zuckerman M. (2021). Phycodnaviruses (*Phycodnaviridae*). Encyclopedia of Virology.

[8] DeLong J.P., Al-Ameeli Z., Lyon S., Van Etten J.L., Dunigan D.D. (2018). Size-dependent Catalysis of Chlorovirus Population Growth by A Messy Feeding Predator. Microb. Ecol..

[9] Al-Ameeli Z.T., AL-Sammak M.A., DeLong J.P., Dunigan D.D., Van Etten J.L. (2021). Catalysis of Chlorovirus Production by the Foraging of *Bursaria truncatella* on *Paramecia bursaria* Containing Endosymbiotic Algae. Microorganisms.

[10] DeLong J.P., Van Etten J.L., Al-Ameeli Z., Agarkova I.V., Dunigan D.D. (2023). The consumption of viruses returns energy to food chains. Proc. Natl. Acad. Sci. USA.

[11] Milrot E., Mutsafi Y., Fridmann-Sirkis Y., Shimoni E., Rechav K., Gurnon J.R., Van Etten J.L., Minsky A. (2016). Virus–host interactions: Insights from the replication cycle of the large *Paramecium bursaria* chlorella virus. Cell. Microbiol..

[12] Carvalho J.V.R.P., Carlson R.M., Ghosh J., Queiroz V.F., de Oliveira E.G., Botelho B.B., Filho C.A.C., Agarkova I.V., McClung O.W., Van Etten J.L. (2024). Genomics and evolutionary analysis of *Chlorella variabilis*-infecting viruses demarcate criteria for defining species of giant viruses. J. Virol..

[13] Duncan G.A., Dunigan D.D., Van Etten J.L. (2020). Diversity of tRNA Clusters in the Chloroviruses. Viruses.

[14] Rodrigues R.A.L., Carvalho J.V.R.P., Henriques L.R., Dunigan D.D., Van Etten J.L. (2026). Taxonomic update for the giant algal chloroviruses of the family *Phycodnaviridae*. Arch. Virol..

[15] Jeanniard A., Dunigan D.D., Gurnon J.R., Agarkova I.V., Kang M., Vitek J., Duncan G., McClung O.W., Larsen M., Claverie J.-M. (2013). Towards defining the chloroviruses: A genomic journey through a genus of large DNA viruses. BMC Genom..

[16] Rodrigues R.A.L., Queiroz V.F., Ghosh J., Dunigan D.D., Van Etten J.L. (2022). Functional Genomic Analyses Reveal an Open Pan-genome for the Chloroviruses and a Potential for Genetic Innovation in New Isolates. J. Virol..

[17] Filho C.A.D.C., Canto I.L.D.J., Botelho B.B.F., Henriques L.R., Carvalho J.V.R.P., Oliveira E.G.D., Souza S.A.D.C., Souza J.W., Teixeira Lima M., Iani F.C.D.M. (2026). A giant virus in a small lake: Characterization of a new tropical algal virus expands the geographical and genetic diversity of the Brazilian virosphere. J. Virol..

[18] Bolger A.M., Lohse M., Usadel B. (2014). Trimmomatic: A flexible trimmer for Illumina sequence data. Bioinformatics.

[19] Bankevich A., Nurk S., Antipov D., Gurevich A.A., Dvorkin M., Kulikov A.S., Lesin V.M., Nikolenko S.I., Pham S., Prjibelski A.D. (2012). SPAdes: A New Genome Assembly Algorithm and Its Applications to Single-Cell Sequencing. J. Comput. Biol..

[20] Hyatt D., Chen G.-L., LoCascio P.F., Land M.L., Larimer F.W., Hauser L.J. (2010). Prodigal: Prokaryotic gene recognition and translation initiation site identification. BMC Bioinform..

[21] Laslett D., Canback B. (2004). ARAGORN, a program to detect tRNA genes and tmRNA genes in nucleotide sequences. Nucleic Acids Res..

[22] Yutin N., Wolf Y.I., Raoult D., Koonin E.V. (2009). Eukaryotic large nucleocytoplasmic DNA viruses: Clusters of orthologous genes and reconstruction of viral genome evolution. Virol. J..

[23] Grant J.R., Enns E., Marinier E., Mandal A., Herman E.K., Chen C., Graham M., Van Domselaar G., Stothard P. (2023). Proksee: In-depth characterization and visualization of bacterial genomes. Nucleic Acids Res..

[24] Jain C., Rodriguez R.L.M., Phillippy A.M., Konstantinidis K.T., Aluru S. (2018). High throughput ANI analysis of 90K prokaryotic genomes reveals clear species boundaries. Nat. Commun..

[25] Nishimura Y., Yamada K., Okazaki Y., Ogata H. (2024). DiGAlign: Versatile and Interactive Visualization of Sequence Alignment for Comparative Genomics. Microbes Environ..

[26] Emms D.M., Kelly S. (2019). OrthoFinder: Phylogenetic orthology inference for comparative genomics. Genome Biol..

[27] Lechner M., Findeiß S., Steiner L., Marz M., Stadler P.F., Prohaska S.J. (2011). Proteinortho: Detection of (Co-)orthologs in large-scale analysis. BMC Bioinform..

[28] Bastian M., Heymann S., Jacomy M. (2009). Gephi: An Open Source Software for Exploring and Manipulating Networks. Proc. Int. AAAI Conf. Web Soc. Media.

[29] Edgar R.C. (2004). MUSCLE: Multiple sequence alignment with high accuracy and high throughput. Nucleic Acids Res..

[30] Capella-Gutiérrez S., Silla-Martínez J.M., Gabaldón T. (2009). trimAl: A tool for automated alignment trimming in large-scale phylogenetic analyses. Bioinformatics.

[31] Minh B.Q., Schmidt H.A., Chernomor O., Schrempf D., Woodhams M.D., von Haeseler A., Lanfear R. (2020). IQ-TREE 2: New Models and Efficient Methods for Phylogenetic Inference in the Genomic Era. Mol. Biol. Evol..

[32] Kalyaanamoorthy S., Minh B.Q., Wong T.K.F., von Haeseler A., Jermiin L.S. (2017). ModelFinder: Fast model selection for accurate phylogenetic estimates. Nat. Methods.

[33] Letunic I., Bork P. (2024). Interactive Tree of Life (iTOL) v6: Recent updates to the phylogenetic tree display and annotation tool. Nucleic Acids Res..

[34] Quispe C.F., Sonderman O., Seng A., Rasmussen B., Weber G., Mueller C., Dunigan D.D., Van Etten J.L. (2016). Three-year survey of abundance, prevalence and genetic diversity of chlorovirus populations in a small urban lake. Arch. Virol..

[35] Henriques L.R., Botelho B.B.F., Carlson R.M., Carvalho J.V.R.P., Oliveira E.G., Agarkova I.V., Van Etten J.L., Dunigan D.D., Rodrigues R.A.L. (2025). Revealing the hidden diversity of *Chlorella heliozoae*-infecting giant viruses. npj Viruses.

[36] Iyer L.M., Makarova K.S., Koonin E.V., Aravind L. (2004). Comparative genomics of the FtsK–HerA superfamily of pumping ATPases: Implications for the origins of chromosome segregation, cell division and viral capsid packaging. Nucleic Acids Res..

[37] Chelikani V., Ranjan T., Kondabagil K. (2014). Revisiting the genome packaging in viruses with lessons from the “Giants”. Virology.

[38] Van Etten J.L., Agarkova I.V., Dunigan D.D. (2020). Chloroviruses. Viruses.

[39] Cho H.-H., Park H.-H., Kim J.-O., Choi T.-J. (2002). Isolation and Characterization of Chlorella Viruses from Freshwater Sources in Korea. Mol. Cells.

[40] Bubeck J.A., Pfitzner A.J.P. (2005). Isolation and characterization of a new type of chlorovirus that infects an endosymbiotic Chlorella strain of the heliozoon *Acanthocystis turfacea*. J. Gen. Virol..

[41] Agarkova I., Hertel B., Zhang X., Lane L., Tchourbanov A., Dunigan D.D., Thiel G., Rossmann M.G., Van Etten J.L. (2014). Dynamic attachment of Chlorovirus PBCV1 to *Chlorella variabilis*. Virology.

[42] Agarkova I.V., Lane L.C., Dunigan D.D., Quispe C.F., Duncan G.A., Milrot E., Minsky A., Esmael A., Ghosh J.S., Van Etten J.L. (2021). Identification of a Chlorovirus PBCV-1 Protein Involved in Degrading the Host Cell Wall during Virus Infection. Viruses.

[43] Zhang Y., Nelson M., Nietfeldt J., Xia Y., Burbank D., Ropp S., Van Etten J.L. (1998). Chlorella Virus NY-2A Encodes at Least 12 DNA Endonuclease/Methyltransferase Genes. Virology.

[44] Coy S.R., Gann E.R., Papoulis S.E., Holder M.E., Ajami N.J., Petrosino J.F., Zinser E.R., Van Etten J.L., Wilhelm S.W. (2020). SMRT Sequencing of *Paramecium bursaria* Chlorella Virus-1 Reveals Diverse Methylation Stability in Adenines Targeted by Restriction Modification Systems. Front. Microbiol..

[45] Van Etten J.L., Schuster A.M., Girton L., Burbank D.E., Swinton D., Hattman S. (1985). DNA methylation of viruses infecting a eukaryotic Chlorella -like green alga. Nucleic Acids Res..

[46] Nelson M., Burbank D.E., Van Etten J.L. (1998). Chlorella Viruses Encode Multiple DNA Methyltransferases. Biol. Chem..

[47] Shields S.L., Burbank D.E., Grabherr R., Van Etten J.L. (1990). Cloning and sequencing the cytosine methyltransferase gene M.CviJI from Chlorella virus IL3A. Virology.

[48] Stefan C., Xia Y., Van Etten J.L. (1991). Molecular cloning and characterization of the gene encoding the adenine methyltransferase M. C viRI from Chlorella virus XZ6E. Nucleic Acids Res..

[49] Dickey J.S., Van Etten J.L., Osheroff N. (2005). DNA Methylation Impacts the Cleavage Activity of Chlorella Virus Topoisomerase II. Biochemistry.

[50] Lyko F. (2018). The DNA methyltransferase family: A versatile toolkit for epigenetic regulation. Nat. Rev. Genet..

[51] Agarkova I.V., Dunigan D.D., Van Etten J.L. (2006). Virion-Associated Restriction Endonucleases of Chloroviruses. J. Virol..

[52] (2025). New England Biolabs. https://www.neb.com/en/search#q=chlorella%20virus&sort=relevancy.

