# Supplementary material for: A Novel Brazilian Isolate Expands the Genomic and Phenotypic Characterization of Chlorovirus americanus
Source: Viruses. 2026 Oct 9;18(10):1119. doi: 10.3390/v18101119 (PMC13709665; doi:10.3390/v18101119)
Supplement: Supplementary file 1 [file viruses-18-01119-s001.zip › viruses-4576708-supplementary.pdf]

SUPPLEMENTARY MATERIAL

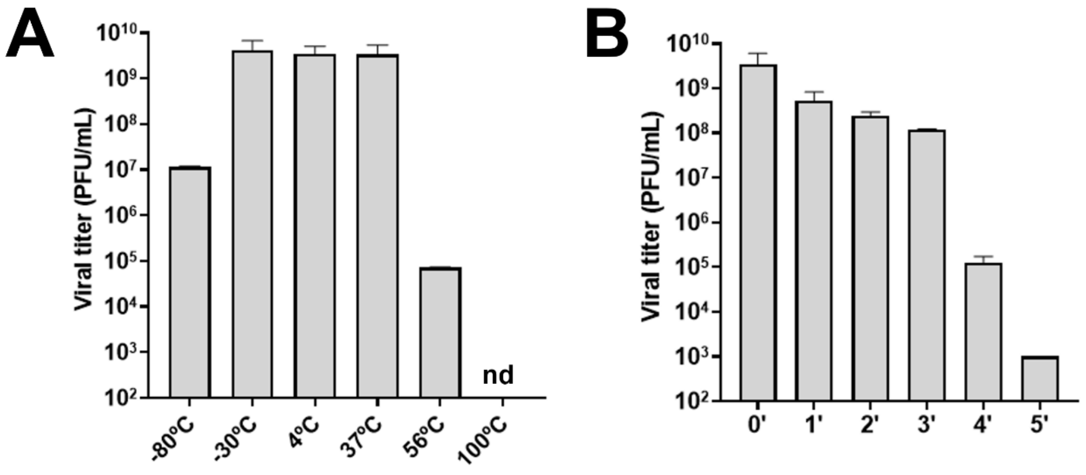

Figure S1. Viral resistance to environmental factors. A) Temperature resistance of BR-MG-N02; B) UV radiation sensitivity of BR-MG-N02. Biological assays were performed in duplicate. nd: not detected.

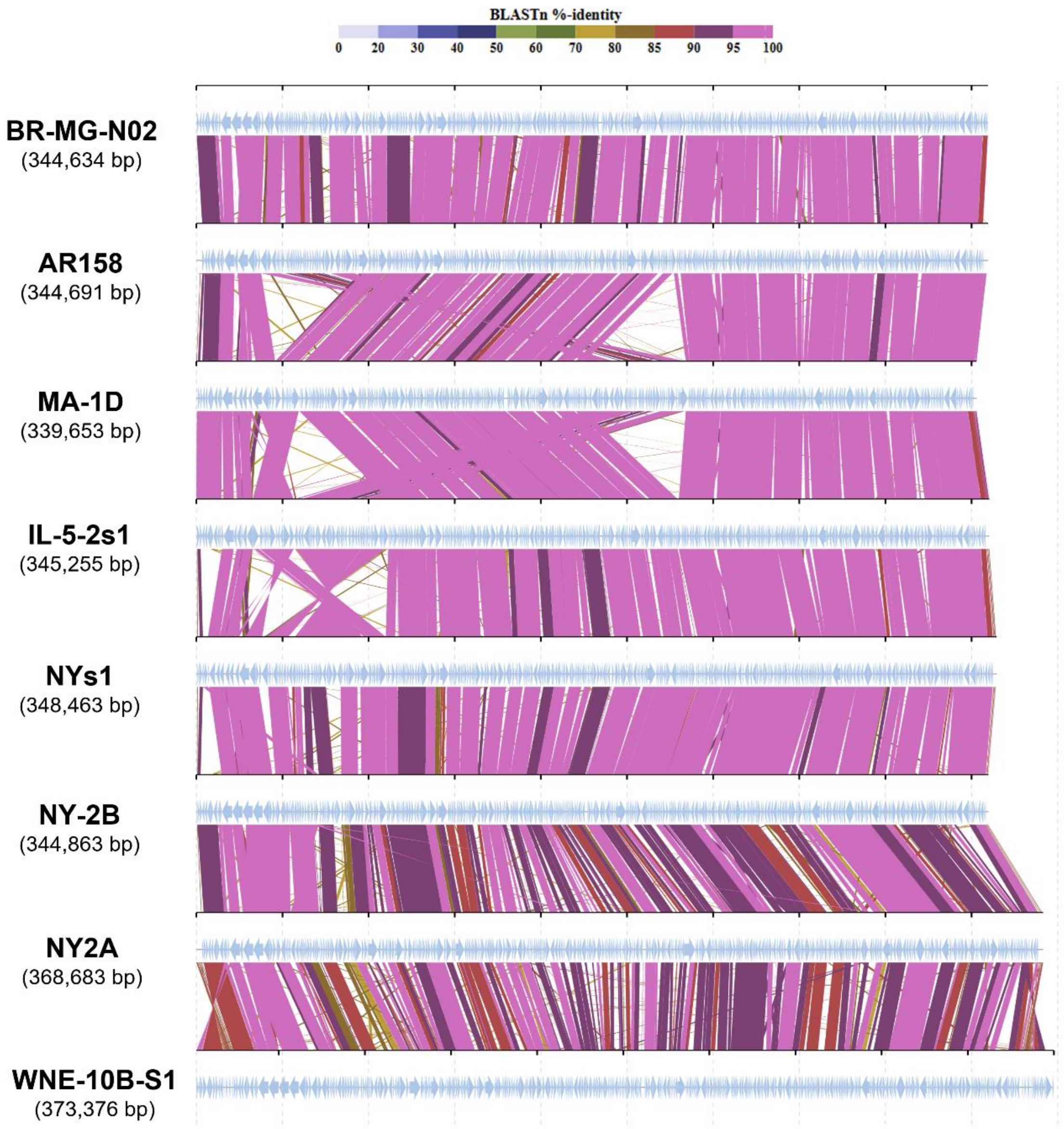

Figure S2. Genome synteny of *Chlorovirus americanus*. Viral genomes were aligned, and colored lines represent BLASTn identity values. The name of the isolates and their genome sizes are indicated on the left side of the respective genome representation

Table S1: General information about *Chlorovirus americanus* isolates.

Virus	Genome size (bp)	CDS count	tRNA count	GC content	Location of isolation	Year of description	GenBank accession
**BR-MG-N02**	344634	389	11	40.7%	Minas Gerais, Brazil	2026	PV975788
**AR158**	344691	388	7	40.76%	Buenos Aires, Argentina	2007	NC_009899
**MA-1D**	339653	368	11	40.69%	Massachusetts, USA	2013	JX997172
**IL-5–2s1**	345255	377	8	40.19%	Illinois, USA	2013	JX997170
**NY-2B**	344863	370	8	40.53%	New York, USA	2013	JX997182
**NYs1**	348463	378	8	40.71%	New York, USA	2013	NC_043235
**NY2A**	368683	412	7	40.69%	New York, USA	2007	NC_009898
**WNE-10B-S1**	373376	403	11	40.87%	Nebraska, USA	2024	PP681875

Table S2: Diversity of tRNA in *Chlorovirus americanus*.

Virus	Islet start (nt)*	Islet end (nt)*	Genome position	Island size (nt)	Leu (taa)	Leu (caa)	Ile (tat)	Asn (gtt)	Arg (tct)	Gly (tcc)	Lys (ctt)	Lys (ttt)	Gln (ctg)	Val (aac)
**NY-2B**	**169407**	**170240**	**49%**	**833**	1	1	1	1	1	1	1			1
**IL-5-2s1**	**175844**	**176677**	**51%**	**833**	1	1	1	1	1	1	1			1
**NY2A**	**194698**	**195555**	**53%**	**857**	1	1		1	1	1	1			1
**NYs1**	**180325**	**182145**	**52%**	**1820**	1	1	1	1	1	1	1			1
**AR158**	**172099**	**172781**	**50%**	**682**	1	1	1	1	1	1				1
**MA-1D**	**134220**	**135350**	**40%**	**1130**	1	1	1	2	1	1	1	1	1	1
**WNE-10B-S1**	**193976**	**195106**	**52%**	**1130**	1	1	1	2	1	1	1	1	1	1
**BR-MG-N02**	**175386**	**176516**	**51%**	**1130**	1	1	1	2	1	1	1	1	1	1

*Genome coordinates may vary from information available in GenBank due to reorganization in orientation to allow proper comparison.
